# Programmable RNA editing with endogenous ADAR enzymes – a feasible option for the treatment of inherited retinal disease?

**DOI:** 10.3389/fnmol.2023.1092913

**Published:** 2023-05-24

**Authors:** Julia-Sophia Bellingrath, Michelle E. McClements, M. Dominik Fischer, Robert E. MacLaren

**Affiliations:** ^1^Nuffield Laboratory of Ophthalmology, Nuffield Department of Clinical Neurosciences, University of Oxford, Oxford, United Kingdom; ^2^Oxford Eye Hospital, Oxford University Hospitals NHS Trust, Oxford, United Kingdom

**Keywords:** RNA editing, adenine deaminase acting on RNA (ADAR), inherited retinal degeneration (IRD), adeno-associated virus (AAV) vectors, circular guide RNA

## Abstract

RNA editing holds great promise for the therapeutic correction of pathogenic, single nucleotide variants (SNV) in the human transcriptome since it does not risk creating permanent off-targets edits in the genome and has the potential for innovative delivery options. Adenine deaminases acting on RNA (ADAR) enzymes catalyse the most widespread form of posttranscriptional RNA editing in humans and their ability to hydrolytically deaminate adenosine to inosine in double stranded RNA (dsRNA) has been harnessed to change pathogenic single nucleotide variants (SNVs) in the human genome on a transcriptional level. Until now, the most promising target editing rates have been achieved by exogenous delivery of the catalytically active ADAR deaminase domain (ADAR_DD_) fused to an RNA binding protein. While it has been shown that endogenous ADARs can be recruited to a defined target site with the sole help of an ADAR-recruiting guide RNA, thus freeing up packaging space, decreasing the chance of an immune response against a foreign protein, and decreasing transcriptome-wide off-target effects, this approach has been limited by a low editing efficiency. Through the recent development of novel circular ADAR-recruiting guide RNAs as well as the optimisation of ADAR-recruiting antisense oligonucleotides, RNA editing with endogenous ADAR is now showing promising target editing efficiency *in vitro* and *in vivo*. A target editing efficiency comparable to RNA editing with exogenous ADAR was shown both in wild-type and disease mouse models as well as in wild-type non-human primates (NHP) immediately following and up to 6 weeks after application. With these encouraging results, RNA editing with endogenous ADAR has the potential to present an attractive option for the treatment of inherited retinal diseases (IRDs), a field where gene replacement therapy has been established as safe and efficacious, but where an unmet need still exists for genes that exceed the packaging capacity of an adeno associated virus (AAV) or are expressed in more than one retinal isoform. This review aims to give an overview of the recent developments in the field of RNA editing with endogenous ADAR and assess its applicability for the field of treatment of IRD.

## Introduction

The eye has been at the forefront of innovative genetic therapies due to its relative accessibility, ease of drug administration that precludes systemic administration of therapeutics as well as the availability of functional outcome measures for therapeutic evaluation. Inherited retinal diseases (IRDs) in particular have been at the forefront of diseases for which gene therapies are currently being developed. There is an imperative need for therapies to cure IRDs: they have a world-wide prevalence of about 1 in 2000, affect more than two million people and cause significant economic as well as and psychological burden (Liew et al., 2014; Chaumet-Riffaud et al., 2017). While gene replacement therapy has proven to be a safe and effective therapy for IRDs, with an approved treatment for *RPE-65*-associated IRD now available and others being evaluated in clinical trial (Russell et al., 2017; Vazquez-Dominguez et al., 2019), an unmet therapeutic need remains for genes too large to fit into the 4.7 kB packaging constraints of an Adeno-associated virus (AAV) (e.g., *ABCA4 or USH2A*), the gold standard delivery vehicle for most gene replacement therapies, or genes that are expressed as multiple isoforms in the human retina (e.g., *CRB1*) (Bellingrath et al., 2021; Fry et al., 2021).

RNA therapeutics work by reversibly changing the transcriptomic sequence without inducing permanent genomic changes, thus eschewing the risk of udesired, permanent genomic off-target effects of DNA editing techniques (Carroll, 2019). RNA therapeutics can broadly be classified into antisense oligonucleotides (ASOs), RNA interference (RNAi) and RNA editing therapies. ASOs are currently under clinical investigation for treatment of several IRDs, such as *CEP290*- and *USH2A*-associated IRDs (Xue and MacLaren, 2020). RNAi therapeutics for IRDs are being developed in preclinical stages with the primary therapeutic target of autosomal dominant RP (adRP), where a dual RNAi suppression strategy using artificial mirtrons, atypical RNA interference effectors spliced from transcripts as short introns, is coupled with a gene replacement strategy (Orlans et al., 2021). For a comprehensive review on RNA therapeutics and their use in the eye the review by Kumar et al. (2022) is recommended. The third RNA therapeutic is RNA editing, which is derived from an essential post-transcriptional modification in humans. The most common form of RNA editing in humans involves the hydrolytic deamination of adenosine (A) to inosine (I) at the C6 position (A-to-I editing) by a family of enzymes aptly named Adenosine deaminase acting on RNA (ADARs) (Bass and Weintraub, 1988; Wagner et al., 1989; Bass, 2002; Nishikura, 2016). The catalytically active ADAR enzymes, ADAR1 and ADAR2, share a C-terminal deaminase domain (ADAR_DD_) and have multiple double stranded RNA binding domains (dsRBD), with which they bind to double stranded RNA (dsRNA) and edit tens of thousands to millions of sites in the human transcriptome (Bazak et al., 2014; Picardi et al., 2015; Tan et al., 2017). When A-to-I deamination takes place in coding sequences, inosine is biochemically read as guanine by the translational and splicing machinery due to their structural similarity and inosine is paired with cytosine during translation (Bass, 2002; Nishikura, 2010). While only the minority of A-to-I editing takes place in coding sequences, it is a powerful and essential tool for proteome diversification, particularly in neurons, where A-to-I editing mediated protein recoding modulates receptor and ion channel properties (Jepson and Reenan, 2008; Hood and Emeson, 2012; Rosenthal and Seeburg, 2012). RNA editing harnesses ADAR’s recoding ability and uses it as a programmable tool to correct pathogenic single nucleotide variants (SNVs).

Therapeutic RNA editing can broadly be divided into approaches utilising exogenous or endogenous ADAR as catalytically active deamination effectors. The first strategy facilitates editing by overexpression of the either full-length ADAR protein or, more commonly, overexpression of the catalytically active deaminase domain fused to an intermediate RNA binding protein such as deactivated Cas13b (dCas13b) (Cox et al., 2017). The second strategy aims to harness the cell’s native, endogenously expressed ADAR enzymes and recruit these for deamination. Both exogenous and endogenous ADAR editing approaches require a programmable guide RNA (gRNA) that is antisense to the target sequence and marks the target adenosine with an A-C mismatch. The gRNA and the target RNA sequence create a dsRNA that serves as a substrate for the ADAR deamination. For all exogenous ADAR approaches harnessing an intermediate binding protein, the gRNA carries a recruitment sequence that binds the intermediary protein. For approaches harnessing the endogenous ADAR or recruiting an exogenously expressed full-length ADAR, the gRNA either carries an ADAR recruitment sequence or simply relies on the dsRNA structure to recruit ADAR. Like other genome editing approaches, the central challenge is to design a system that achieves deamination with high on-target editing efficiency and specificity for the target adenosine, while minimising bystander and transcriptome-wide off-target edits. RNA editing with exogeneous ADAR has exhibited high on-target editing efficiency but has been hampered with high transcriptome-wide off-target editing. Therapeutic endogenous ADAR RNA editing has up until now been unable to compete with exogenous RNA editing due to low on-target efficiency. However, due to exciting developments in the endogenous RNA editing field which succeed in stabilising the gRNA by circularisation or chemical modification, endogenous ADAR editing might be approaching viability in an *in vivo*, therapeutic clinical setting. Whether this is relevant for ocular gene editing will depend on the ADAR-tissue expression as well as finding a suitable vehicle for delivery.

To unlock the utility of ADARs as an RNA editing toolset, editing must be achieved with a high on-target efficiency and specificity which precludes off-target editing in the form of bystander editing and transcriptome-wide off-target editing. For editing with endogenous ADAR, the central challenges are improving on-target editing efficiency while limiting bystander editing in the gRNA:target RNA duplex, while the central challenge for exogenous ADAR editing is restricting promiscuous transcriptome-wide off target editing and achieving target specificity while retaining high on target editing rates.

This review aims to give an outline of the history and recent developments in the field of RNA editing with endogenous ADARs and discuss the potential for treatment of IRDs. Since an understanding of the structure, function and role of ADAR enzymes is essential to understanding the design, challenges and strategies in the RNA editing field, an overview of ADAR enzymes will proceed the discussion on RNA editing with endogenous ADAR.

### Enzyme structure, function, and mechanism of ADAR editing

#### ADAR protein domains, isoforms, cellular localisation, and tissue expression

##### The ADAR family has two catalytically active members which share protein domains and differ in their cellular localisation and tissue expression

**Figure 1:**
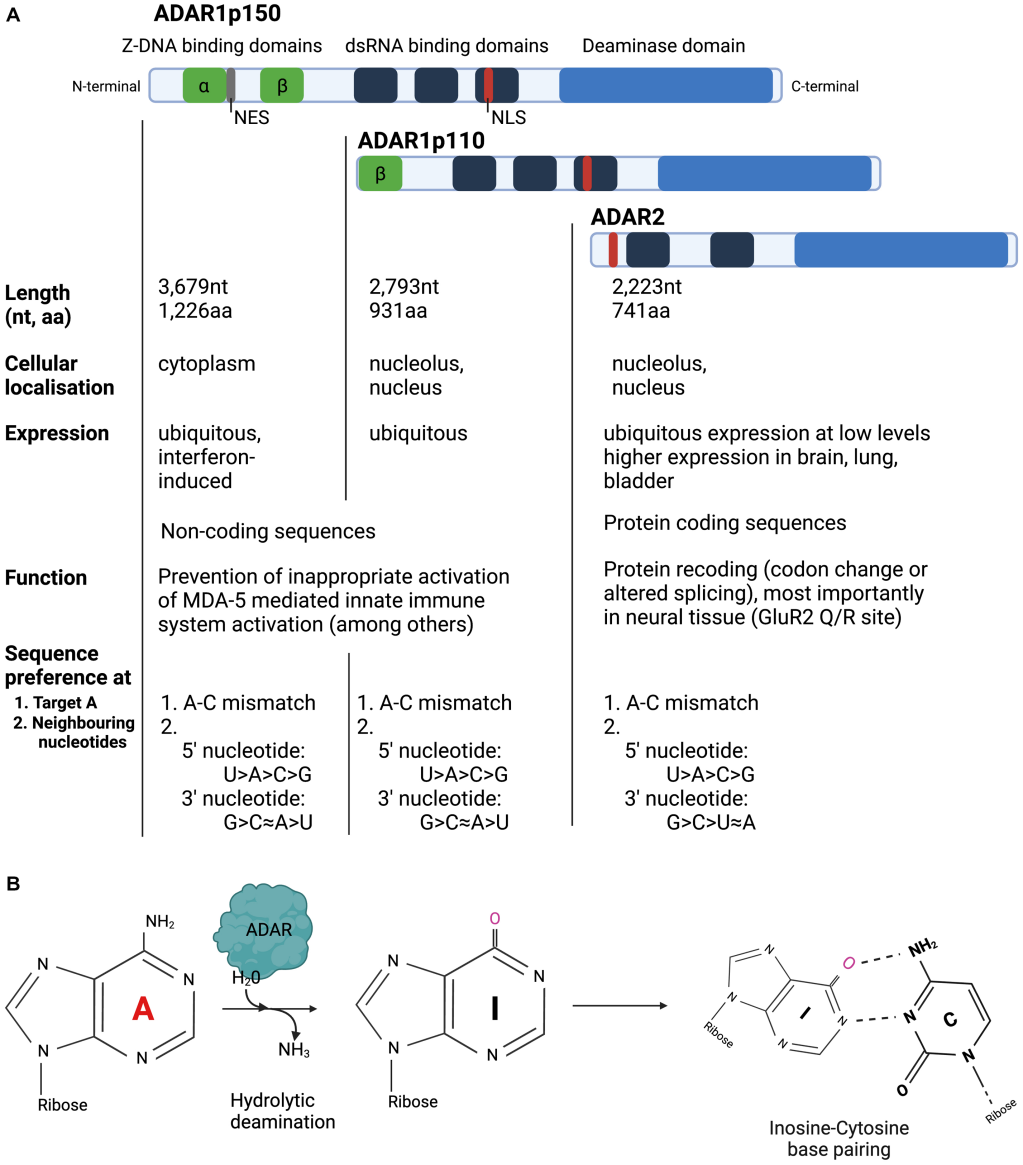
Overview of structure and function of ADAR enzymes. **(A)** ADARp110 and ADAR2 are expressed predominantly in the nucleolus and nucleus, while ADAR1p150 is expressed in the cytoplasm. ADAR1p110 is expressed ubiquitously at high levels and is primarily responsible for editing in noncoding regions. ADAR1 expression is ubiquitous, whereas ADAR2 expression is highest in the brain, bladder, and lung. **(B)** ADAR enzymes hydrolytically deaminate Adenosine to Inosine in dsRNA. Inosine is biochemically read as Guanosine.

Three members of the ADAR family are encoded in the mammalian genome and are highly conserved across species (Slavov et al., 2000): ADAR1, ADAR2 and ADAR3 (Kim et al., 1994; Melcher et al., 1996a,b; Chen et al., 2000). ADAR1 is expressed as two separate isoforms: the full-length ADAR1p150, which is under the control of an interferon alpha (IFN-alpha) inducible promotor and the shorter ADAR1p110 isoform, which is constitutively expressed (Patterson and Samuel, 1995; Strehblow et al., 2002). ADARs from all characterised species have two main structural motifs: several double stranded RNA Binding Domains (dsRBD) and a single Deaminase Domain (DD) (Figure 1; Stefl et al., 2006). Each dsRBD is approximately 65 amino acids (aa) in length and makes direct contact with dsRNA, recognising higher order structures within dsRNA. While dsRBDs bind perfectly matched duplex RNA, they can also bind imperfectly matched structures with bulges, hairpins, and mismatches with high affinity (Polson et al., 1996; Bass, 2002; Montiel-Gonzalez et al., 2019). Both ADAR1 isoforms and ADAR2 have a single deaminase domain (DD) at their carboxy (C-) terminus that form the enzyme’s catalytic centre. ADAR1 has unique Z-DNA binding domains (ZBD), that have been shown to bind both Z-DNA and Z-RNA, but whose function remains poorly understood (Herbert et al., 1997; Brown et al., 2000). The full-length ADAR1p150 isoform contains two ZBDs, ZBD alpha and beta. ZBD alpha domain is only present in ADAR1p150, whereas the shorter ADAR1p110 only has one ZBD, Z beta, at its N-terminus. Both ADAR1 isoforms contain a Nuclear Localisation Signal (NLS) in the third dsRBD, but only ADAR1p150 contains a Nuclear Export Signal (NES), which is located in the ZBD alpha domain. ADAR2 contains a NLS, located toward the N-terminus. ADARp150 can shuttle from the nucleus to the cytoplasm, but in accordance with the NES found in its ZBD beta, ADAR1p150 is detected mainly in the cytoplasm (Patterson and Samuel, 1995). ADARp110 and ADAR2 are expressed predominantly in the nucleolus and nucleus (Desterro et al., 2003). ADAR1p110 is expressed ubiquitously at high levels and is therefore responsible for the majority of editing activity, particularly in repetitive sequences in noncoding regions, which are particularly suited for dsRNA formation due to their inherent ability to base pair with themselves (Mannion et al., 2015; Tan et al., 2017; Walkley and Li, 2017; Eisenberg and Levanon, 2018). While ADAR2 expression is ubiquitous, it is generally expressed at much lower levels than ADAR1. The exception to this is ADAR2 expression in the brain, bladder, and lung, which exhibit high levels of ADAR2 expression (The Human Protein Atlas^1^).

The presence of ADAR2 and ADAR1 protein expression levels in the neuronal cell types of the retina has not yet been comprehensively analysed, but immunohistochemical evidence points toward ADAR2 expression in the retinal ganglion cell (RGC) layer (Wang et al., 2014). It is also unclear how much protein ADAR protein expression is needed to induce therapeutic editing in the retina. In concordance with its expression profile, ADAR2’s protein recoding ability is particularly important in the neuronal system (Li et al., 2009; Picardi et al., 2015). ADAR3 has a catalytically inactive deaminase domain, its expression is restricted to the brain and is thought play an inhibitory role of RNA editing by the catalytically active ADARs (Chen et al., 2000; GTEx Consortium, 2013). Due to its lack of A-to-I editing, it will not be the focus of this review.

#### Effect and function of A-to-I editing

##### ADAR-mediated A-to-I editing takes place in coding and non-coding regions of the genome and has an array of different functions that can be harnessed for programmable, therapeutic RNA editing

In general, ADARs mediate A-to-I editing in both coding regions, where editing can lead to proteome diversification through codon changes and induction of alternative splicing, and in non-coding regions, where one of the many functions comprises the modulation of the innate immune response (Bass, 1997; Bass, 2000; Nishikura, 2010; Mannion et al., 2015; Nishikura, 2016; Walkley and Li, 2017; Eisenberg and Levanon, 2018). Certain transcript targets are edited exclusively by ADAR1 or ADAR2, whereas other sites can be edited by both (Nishikura, 2016, tables 1 and 2) (Lehmann and Bass, 2000; Nishikura, 2016). ADAR2 is the primary, but not the exclusive editor of protein coding sequences in mammals (Lehmann and Bass, 2000; Tan et al., 2017). Through this A-to-I mediated alterations of coding sequences and splicing sequences, ADARs are capable of creating different protein isoforms as well as altering and regulating gene expression at an RNA level. Two of the most well-characterised ADAR editing sites are the R/G and the Q/R site of the pre-mRNA encoding the subunits of the a-amino-3-hydroxyl-5-methyl-4-isoxazole-propionate (AMPA)- subtype of ionotropic glutamate receptor (GRIA2, also known as GluR2) (Higuchi et al., 1993; Melcher et al., 1996b; Higuchi et al., 2000). Both ADAR1 and ADAR2 can edit the R/G site (Lomeli et al., 1994) and this sequence is of particular importance in RNA editing because of its use as a physiological recruitment sequence for endogenous or full-length exogenous ADAR in the gRNA design (Fukuda et al., 2017; Wettengel et al., 2017; Katrekar et al., 2019; Merkle et al., 2019; Katrekar et al., 2022; Reautschnig et al., 2022). The ADAR dsRBDs bind to the pre-mRNA exon/intron border at the R/G site of the GluR2 receptor and results in highly specific ADAR-mediated editing at this site (Stefl et al., 2010). Mouse knockout studies suggest that the GluR2 Q/R recoding site is ADAR2’s only essential target (Higuchi et al., 2000), where it converts the CAG (Glutamine, Q) codon to CGG (arginine, R) at the Q/R site of the GRIA2 subunit. Adar2-null mice experience neuronal death due to an excess influx of calcium, which leads to frequent epileptic seizures and death several weeks after birth (Higuchi et al., 2000; Horsch et al., 2011). This severe phenotype is fully rescued by introducing an allele containing an arginine (R) codon for the Q/R site in knockout mice. Other physiologically important mammalian genes that undergo recoding type editing that alters their protein function is the G-protein-coupled serotonin receptor 5-HTR2C, the voltage-gated potassium channel Kv1.1 and the alpha-3 subunit of GABA_A_ receptor (Burns et al., 1997; Bhalla et al., 2004; Daniel et al., 2011).

While the recoding of proteins is of most interest when harnessing ADARs for a therapeutic design, it should be stated that vast majority of native A-to-I editing takes place in the non-coding region of the human genome such as introns, untranslated regions and non-coding RNA. Within the non-coding region, editing mostly takes place within mobile elements such as Alu [part of the class of short interspersed elements (SINE)] and long interspersed elements LINEs (Kim et al., 2004). ADAR1 is recognised as the primary editor of these sequences (Tan et al., 2017; Costa Cruz et al., 2020). Over 99% of the editing are detected in Alu repeats (Athanasiadis et al., 2004; Blow et al., 2004; Kim et al., 2004; Levanon et al., 2004), and similarly to the GluR2 motif, Alu elements have therefore been used as recruiting domains for endogenous ADAR (Katrekar et al., 2022). Perhaps the most prominent function of ADAR1 is the suppression of the activation of the innate immune response to endogenous ADAR (Chung et al., 2018). A-to-I editing of long, dsRNA motifs disrupt the perfect pairing of the repetitive RNA transcripts and hinders melanoma differentiation-associated gene 5 (MDA-5) binding and the downstream activation of the interferon induced innate immune response (Chung et al., 2018; Lamers et al., 2019). Murine studies showed the embryonic lethality of *Adar1* knockout mice, likely due to an aberrant interferon response and subsequent stress induced apoptosis of liver haematopoietic cells (Hartner et al., 2004; Wang et al., 2004). In a human knockout neuronal progenitor cell line, MDA5 (dsRNA sensor)-dependent spontaneous interferon production, Protein kinase R (PKR) activation and cell death was observed (Chung et al., 2018). In humans, mutations in ADAR1 have been shown to cause type I interferonopathies such as Aicardi-Goutières-Syndrome (Rice et al., 2012). ADAR1 has many other important physiological functions, such as the altering of miRNA maturation and targeting as well as promoting genome diversification, and roles in human disease pathogenesis. For excellent reviews on this topic, as well the details of ADAR editing in coding and non-coding regions, refer to Nishikura, (2016) and Song et al., (2022).

#### ADAR target specificity is determined by sequence preference and structure selectivity

##### While still incompletely understood, ADAR exhibits a nucleotide sequence preference and a secondary double stranded RNA structural selectivity, which determines target adenosine selectivity

It has yet to be fully understood which characteristics determine whether an adenosine within dsRNA is deaminated, but it is thought that ADAR exhibits a sequence preference for particular RNA motifs (Eggington et al., 2011; Matthews et al., 2016) while the secondary structure of the dsRNA substrate confers editing selectivity (Nishikura et al., 1991; Lehmann and Bass, 1999).

ADAR preferentially deaminates adenosines that occur in an A-C mismatch over those that occur as A-A or A-G mismatches or in an A-U pairing (Wong, 2001). These characteristics are commonly used to confer target specificity in a gRNA when an A-C mismatch is used to mark the adenosine. On the other hand, this preference is used to avoid bystander editing in a long, gRNA:targetRNA duplex by creating an A-to-G mismatch across from adenosines vulnerable to bystander edits (Cox et al., 2017; Abudayyeh et al., 2019; Katrekar et al., 2019; Qu et al., 2019; Yi et al., 2022).

Although ADARs do not exhibit strict sequence specificity, the enzymes do exhibit a preference for certain neighbouring nucleotides. The most pronounced preference is seen for the base flanking the 5′ end of the target adenosine. Here, both ADAR1 and ADAR2 favour Uridine (U) as a neighbouring base, followed by A, C and G (U > A > C > G). While the nature of the 3′ neighbouring base is thought to be less important, both ADARs prefer guanosine (G) at this position. More specifically, ADAR1 prefers G > C ≈ A > U and ADAR2 exhibits a slightly different order of preference: G > C > U ≈ A (Eggington et al., 2011; Matthews et al., 2016). These preferences make the stop codon RNA motif 5′-TAG-3′ a preferential editing site, whereas a particularly difficult RNA motif to edit would be one with a 5′ neighbouring G (5’-GAN-3′).

Beyond sequence-specific preferences, ADAR-dsRNA interactions rely on the structure of the RNA substrate to confer target editing specificity. Generally, ADAR acts on both inter- and intramolecular dsRNA of >20 bp in length (Nishikura et al., 1991; Bass, 1997; Lehmann and Bass, 1999). Long (>100 bp), perfectly matched dsRNAs have been found to be non-selectively deaminated and exhibit an editing rate of 50–60%, whereas shorter dsRNA structures are edited more selectively (editing rate < 10%) indicating that the secondary structure of dsRNA may dictate editing site selectivity (Bass, 1997; Lehmann and Bass, 1999). In general, imperfectly paired dsRNA which is periodically interrupted with mismatches or loops of at least 6 bp exhibited a much more selective deamination than perfectly paired dsRNA, since internal loops are equivalent to helix termini for ADAR1 (Lehmann and Bass, 1999). The non-specific editing of long stretches of dsRNA might be explained by the presence of dsRBD which are thought to bind to dsRNA in a sequence independent manner (Tian et al., 2004). Thus, the insertion of internal loops into long stretches of dsRNA are another strategy to decrease the bystander deamination events that occur in particular when using guide RNAs with a long specificity domain within the dsRNA substrate and confer selectivity in dsRNA deamination (Katrekar et al., 2022).

Since many RNA strategies employ only the deaminase domain of ADAR, it is important to note that ADAR_DD_ alone also has targeting capacities (Phelps et al., 2015). However, our understanding of the features controlling ADAR target recognition is incomplete and a precise prediction of editing sites is not currently possible (Eisenberg and Levanon, 2018). Once the target adenosine has been identified, a base-flipping mechanism enables the target adenosine to access the enzyme’s catalytic site. The ADAR base flipping loop approaches the RNA duplex from the minor groove side and flips the adenosine out of the RNA double helix and into the enzyme’s catalytic pocket, where the hydrolytic A-to-I deamination reaction takes place at the C6 position (Matthews et al., 2016). Structural studies have revealed the presence of inositol hexakisphosphate (INsP6) in the enzyme core and close to the catalytic centre (Macbeth et al., 2005). The deaminase domain can function as an independent catalytic unit in the absence of the dsRBD. The space vacated by the reactive base is stabilised by the intercalation of the E488 amino acid side chain. This amino acid (among others) can be mutated to induce a hyperactive ADAR variant widely employed in RNA editing (Cox et al., 2017; Katrekar et al., 2019).

### RNA editing with endogenous ADAR

#### Constructing a guide RNA capable of recruiting full-length ADAR

In a landmark study almost 30 years ago Woolf et al. showed the potential of endogenous ADAR recruitment for therapeutic RNA editing when they demonstrated A-to-I editing in a Xenopus embryo microinjected with a pre-assembled duplex consisting of a target RNA hybridised to a 52 nt long, unstructured gRNA (Woolf et al., 1995). Two central hurdles of editing with endogenous ADAR, namely low on target efficiency and bystander edits, were already apparent in this landmark study.

In a crucial step toward harnessing endogenous ADAR for RNA editing, Wettengel et al. (2017) designed a gRNA for the recruitment of full-length ADAR in 2017. While this strategy still required the overexpression of full-length ADAR2 under the strong CMV promotor, either from a plasmid or from an ADAR2 expressing cell line, it did not rely on an intermediary protein to mediate gRNA binding to ADAR and thus laid the groundwork for recruiting endogenous ADAR.

The ADAR2-recruiting gRNA was composed of two parts: (1) an antisense sequence complementary to the target RNA substrate located at the 3′ end and (2) the R/G GluR2 ADAR-recruiting domain at the 5′ end. The antisense region contained a C mismatch opposite the target A, and the optimal length was determined to be 18–20 nt. Lengthening of the antisense region beyond this point led to declining editing rates as well as bystander edits.

In an eGFP – reporter plasmid with a premature stop codon, on-target editing rates up to 50% were observed, which increased to 65% when the ADAR2 expression was integrated in the host genome of HEK293T cells under the CMV promotor. When editing components as well as ADAR2 were given at high concentrations, massive off target effects were seen and a gRNA lacking the R/G motif was able to elicit editing. These effects were decreased when lowering the concentrations of all transfected components, indicating that dosing is a central question when trying to achieve desired editing while lowering bystander edits. Furthermore, the authors showed editing of several endogenous housekeeping gene transcripts with editing rates ranging from 10 to 35% in 12 out of 13 target sites. Interestingly enough, one site in beta actin consistently achieved 0% editing, indicating the target variability in editing rates. Apart from showing editing of several endogenous housekeeping genes, a 10% correction of the PINK W437X nonsense mutation, a functional rescue of cellular phenotype was also shown. In a longitudinal analysis of editing, peak editing was observed at 48 h after transfection which remained constant until 72 h and then started to decline at 96 h.

In a follow-up study by Heep et al. (2017) the same gRNA construct was shown to be able to recruit the two inducible as well as the constitutively expressed isoforms of ADAR1 in an ADAR1 expressing cell line. The gRNA design was also optimised to minimise auto-editing of adenine within the R/G GluR2 hairpin by substituting A/U base pairs with C/G base pairs.

Using a very similar gRNA design to Wettengel et al. (2017) and Fukuda et al. (2017) also showed that an ADAR-recruiting gRNA (AD-gRNA) design based on the secondary structure of GluR2 (GRIA2) pre-mRNA, was capable of recruiting ADAR2 in an ADAR2 overexpressing cell line.

#### Lengthening the guide RNA and adding chemical modifications as two strategies of ADAR-recruiting guide RNA optimisation

In 2019, Katrekar et al. used the design of the ADAR-recruiting gRNA (named adRNA after ADAR-recruiting gRNA) described in Wettengel et al. (2017) and Fukuda et al. (2017) and compared delivery of the adRNA alone with the delivery of the adRNA together with full-length exogenous ADAR2 [both wild-type and hyperactive ADAR2 (E488Q)]. Several factors were varied: the length of the antisense region (20, 60, and 100 nt), the number of GluR2 recruiting domains (0, 1, and 2) as well as the position of the A-C mismatch. Several important observations were gleaned from this study: when elongating the adRNA antisense region to 60 bp or more, administration of the adRNA alone (without exogenous ADAR2 overexpression) resulted in editing an endogenously expressed *RABA7* transcript in HEK293T cells through the recruitment of endogenous ADAR. Even when the GluR2 recruitment domains were removed from the adRNA, the adRNA elicited editing. An A-C mismatch placed at the centre of the antisense region exhibited the most effective editing. While the RNA editing through adRNA recruitment of endogenous ADAR exhibited a target editing efficiency lower than the RNA editing achieved with overexpressed wild type and hyperactive ADAR2 (E488Q), this was the first indication that endogenous ADAR was able to be recruited by an ADAR-recruiting gRNA, with and without the ADAR-recruiting domain R/G GluR2. These results held true *in vivo*, when different adRNA designs were tested on a G > A point mutation in a sparse fur ash (*spf^ash^*) mouse model of ornithine transcarbamylase (OTC) deficiency. One month after retroorbital injection adRNA alone resulted in low but significant RNA editing yields in the liver. Nevertheless, the highest edited fraction came from adRNA delivery together with the hyperactive ADAR2 mutations (E488Q) (4.6–33.8% RNA editing).

Building on this work, Qu et al. developed LEAPER (leveraging endogenous ADAR for programmable editing of RNA), an endogenous ADAR-recruiting gRNA (arRNA), consisting of a long, single arRNA without ADAR-recruiting sequences or chemical modifications. During the optimisation of LEAPER, several design principles were established: similar to Katrekar et al. which required an adRNA of at least 60 bp for endogenous ADAR recruitment, a minimum length of at least 71 nt was required for efficient endogenous ADAR recruitment and subsequent editing. The length of the arRNA correlated positively with the target editing efficiency, with arRNA counting 111 and 151 nt being used in experiments in standard and primary cell lines. A mismatch position placed in the middle of the arRNA lead to the highest editing efficiency and the preferred 5′ neighbouring base was 5’U. The standard cell lines all lacked ADAR2 expression, indicating that ADAR1 was being recruited for editing. But in a knockout cell line, editing was rescued by the addition of ADAR2 as well as ADAR1 isoforms, indicating that LEAPER can recruit ADAR1 and ADAR2. In addition to a testing in a reporter plasmid assay, where arRNA achieved a target efficiency of approximately 13%, LEAPER was tested on an array of standard and primary cell lines, showing a wide range of target editing rates. The highest editing rates were shown in the primary bronchial epithelial cells targeting transcripts of the endogenous *PPIB* gene with editing rates >80%. When targeting this same transcript with lentiviral transduction in HEK293T cells, the editing rates dropped to 6% at 6 days post transduction. This efficiency increased to 20% when chemical modifications were introduced in the form of 2-O-methyl and phosphorothioate backbone modifications at either end of the arRNA. This highlights the wide range of editing rates in human primary cell lines, that are dependent on target sequence, delivery mechanism and endogenous ADAR expression in the target tissue.

Due to the length of the antisense domain, As occurring in the target sequence were promiscuously deaminated, although the extent of this varied between targets. The highest bystander editing was seen in the *KRAS* gene transcript, where one-third of As were subject to bystander editing. In an attempt to curb this bystander editing, A to G mismatches were placed across from the potential target As. Clinical relevancy of LEAPER was shown by editing the tumour suppressor gene transcript *TP53* as well as a nonsense mutation in the *IUDA* gene transcript in a primary cell line isolated from a Hurler syndrome patient. Editing rates were modest but nevertheless significant. Expression levels of targeted transcripts were monitored to rule out possible RNA interference (RNAi) effect of the arRNA.

In the same year, Merkle et al. chemically modified their previously described (Wettengel et al., 2017) ADAR2-recruiting gRNA to develop the ADAR-recruiting antisense oligonucleotide construct RESTORE (recruiting endogenous ADAR to specific transcripts for oligonucleotide-mediated RNA editing). Chemical modifications included selective phosphorothioate (PS) backbone stabilisation as well as 2’-O-methyl (2’-OMe) modifications and were intended to stabilise the gRNA and thus enable more efficient recruitment of endogenous ADAR without the need for lengthening the antisense domain subsequent increase of probability for bystander editing. The RESTORE construct consists of a short (20 or 40 nt), programmable antisense specificity domain with an invariant ADAR-recruitment domain based on the naturally occurring R/G motif of the GluR2 subunit, a well-characterised editing target for both ADAR1 and ADAR2 (Lomeli et al., 1994). While the genetically encoded gRNA described in Wettengel et al. (2017) was only able to recruit overexpressed ADAR, the heavily chemically modified ASO achieved significant editing rates in a range of standard and primary cell lines. RESTORE editing relied primarily/heavily on the ADARp150 isoform, since editing rates consistently improved by 2- or 3-fold after IFN-alpha treatment. The two best performing ASO versions, ASOv9.5 and ASOv25, both contained a fully chemically modified R/G GluR2 domain and a chemically modified antisense domain either 18 bp (ASOv9.5) or 40 bp (ASOv25) in length. In ASOv25, locked nucleic acids (LNA) were also included at the 3′ end of the antisense region. ASO9.5 showed 19 and 32% editing in a panel of human standard cells and human primary cell lines, respectively. Since editing relied heavily on ADARp150, IFN-alpha treatment increased these editing rates by 2- or 3-fold. In HeLa cells, ASOv25 showed higher editing of 26% without IFN-alpha and 43% with IFN-alpha. In primary cell lines with the 5′ stop codon of the endogenously expressed GAPDH as a target, editing levels ranged between 9 and 27%, with IFN alpha treatment increasing the target rate by two-fold. To illustrate therapeutic potential of RESTORE *in vitro*, the E342K missense mutation (PiZZ mutation) in the *SERPINA* gene, the most common cause for alpha1 antitrypsin deficiency, was targeted. In a HeLa cell line expressing mutated *SERPINA,* cDNA editing rates of 10% were found without IFN-alpha, which increased to 20% with IFN-alpha treatment. When targeting the Tyr701 site in the 5′ UAU codon of the endogenous STAT1 in HeLa and primary cells, the highest editing yields were achieved with IFN-alpha induction and ranged from 20 to 30%. Neither LEAPER nor RESTORE were tested *in vivo*.

In 2022, the Stafforst group further modified their original gRNA design and created CLUSTER, which retained the basic design principle of RESTORE, but achieved recruitment of endogenous ADAR without chemical modification. While the short, 20 nt antisense target specific domain as well as the invariant GluR2 recruitment domain remained, a series of single stranded RNA recruitment sequences (RS), a “cluster,” were added to the gRNA design. These RS bind to the target mRNA in various regions distal to the target site and distal to each other. These RS were 7–20 nt in length and could be flexibly chosen to exclude adenines that would otherwise be prone to bystander editing. The spacer regions between the RS binding sites were generally between 10 and 460 nt. The Stafforst group provides an open-source bioinformatics tool named recruitment cluster finder (RCF) to enable users a customised CLUSTER gRNA design. This strategy allowed for an increased binding affinity of the gRNA to the target sequence by adding RS while retaining the minimised chance of bystander editing that occurs when using a short specificity domain. Another important difference to RESTORE was the type of ADAR recruited. RESTORE preferentially recruited ADARp150 for editing and thus required IFN-alpha induction, but CLUSTER preferentially recruited the ubiquitously expressed ADARp110 and was also able to show recruitment of ADAR2. In contrast to LEAPER, CLUSTER was shown to benefit from the GluR2 ADAR-recruiting domain, but not from an extension of the specificity domain, so the specificity domain was kept at 20 nt to avoid extraneous bystander editing. At least 2 RS were needed to enable significant editing through the recruitment of endogenous ADAR. A luciferase reporter assay in HeLa cells was used for CLUSTER gRNA optimisation and testing of ADAR-specific recruitment. In HeLa cells using transfection of disease relevant transcripts and CLUSTER gRNA, editing yields between 3 and 61% without bystander edits were observed. When comparing CLUSTER to LEAPER, LEAPER outperformed CLUSTER by 20% on the *BMPR* target transcript, but CLUSTER yielded higher on target efficiencies for the *mIDUA* target transcript. In contrast to LEAPER, which showed high bystander editing rates up to 50%, CLUSTER did not elicit any detectable bystander edits. Coding and non-coding regions of endogenous transcripts with varying levels of expression were targeted in HEK293Ft cells and yielded on target rates of 19–44% without bystander editing. Encouragingly, editing efficiencies were similar for targets in the ORF and the 3’UTR. Fibroblasts taken from a Hurler patient were treated with CLUSTER gRNA in the form of chemically stabilised ASO. Editing rates of 24% were seen as well as an increase in IDUA enzyme activity. Off-target effects were investigated and found to be mainly located in repetitive Alu sequences with three novel exonic off target effects also observed. Of note, Adenovirus (AV) not AAV was used. Finally, two different CLUSTER designs were tested in wild-type C57/BL6 mice by co-delivering the dual luciferase reporter plasmid with a CLUSTER gRNA into the liver by hydrodynamic tail vein injection. Both gRNAs had a similar design with a 20 nt target specificity domain, an invariant GluR2 ADAR-recruiting sequence and 3 × RS. The length of the RS varied between the two constructs with the first having 15-, 13-, and 11 nt and the second gRNA having 20-, 15-, and 15 nt. 72 h post-injection, the first CLUSTER gRNA demonstrated 5% editing rate both in luminescence detection and Sanger sequencing while the second gRNA demonstrated a 10% editing rate.

#### Stabilisation of the guide RNA through circularisation

**Figure 2:**
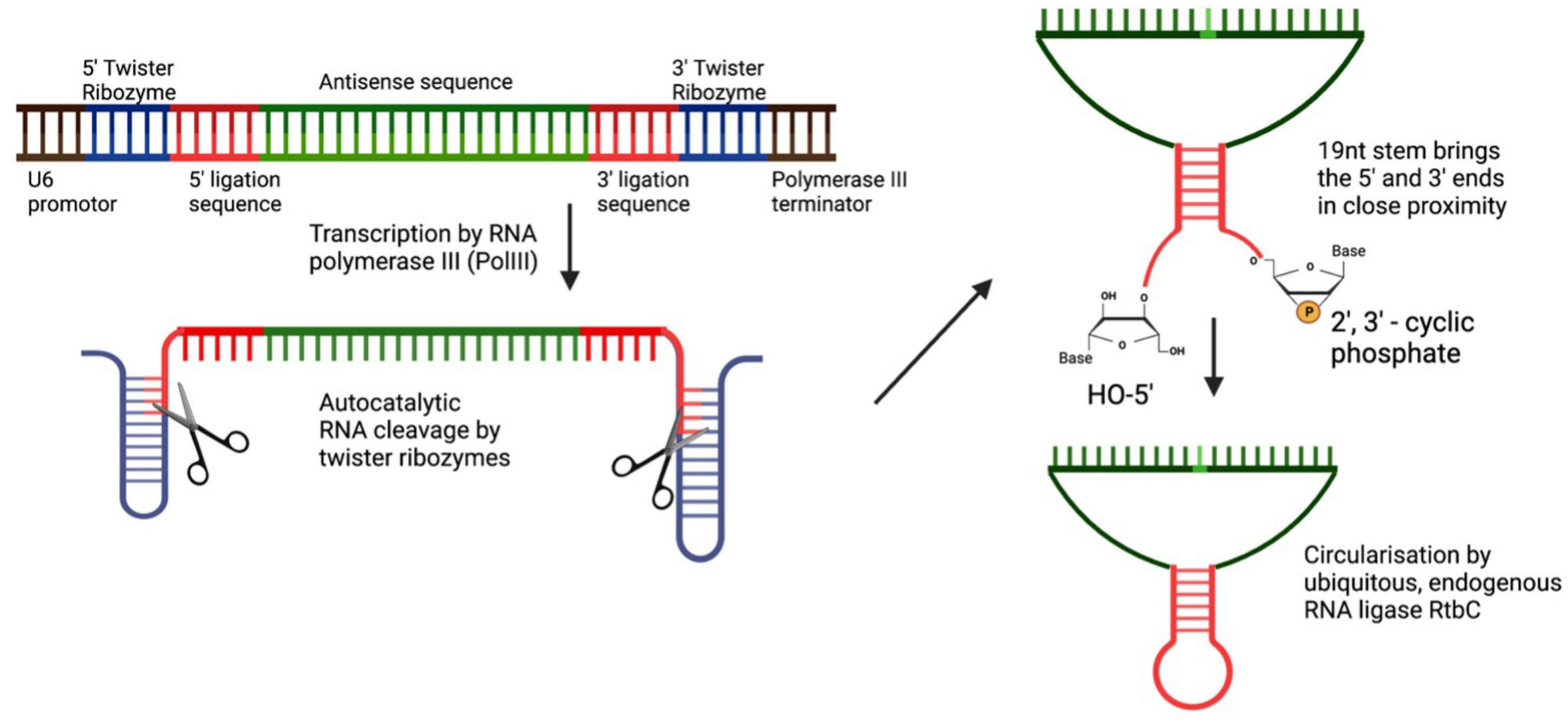
Circularisation of endogenous ADAR using autocatalytic twister ribozymes. Twister ribozymes rapidly self-cleave after transcription has occurred. RNA hybridization brings the unique 5’ and 3’ ends in close proximity for the ligation by the ubiquitously expressed RNA ligase RtbC.

In 2022, two groups utilised the elegant method of RNA circularisation, developed by Litke and Jaffrey (2019) for therapeutic RNA delivery, and applied it to the ADAR-recruiting guide RNA with the idea of enabling a more stable, long-lasting gRNA expression and thus allowing for more efficient on-target editing. Litke based the Tornado (Twister-optimised RNA for durable overexpression) expression system on the endogenous mechanism of RNA circularization in a subset of intron-containing tRNAs, in which a tRNA specific endonuclease (TSEN) cleaves the intronic sequence, thus creating an exonic and intronic sequence that both have unique 5’hydroxy and 2′3’-cyclic phosphate ends. The nearly ubiquitous RNA ligase RtcB uses these unique 5′-and 3′-prime RNA ends to form a mature tRNA from the exonic sequence and circularises the intronic sequence. To produce ribozyme-assisted circular RNA (racRNA), a genetically encoded RNA sequence is flanked by autocatalytic twister ribozymes, which mimic processing by the endogenous TSEN and rapidly self-cleave to produce the unique 5′ and 3′ ends required for circularisation. A short, 19-nucleotide stem was created through RNA hybridisation and this stem placed the unique 5′ hydroxyl and 2′-3′ phosphate 3′ ends near each other to facilitate recognition and ligation by RtcB RNA ligase (Figure 2). Since ADAR-recruiting gRNA are usually transcribed from a polIII promoter such as U6, they do not have a 5′ prime cap and polyA tail, which makes them prone to rapid degradation by cellular exonucleases. The ring structure of circular RNA protects against exonuclease-mediated degradation and thus confers circular RNA with a high stability and consequently, a longer half-life (Litke and Jaffrey, 2019). The ribozyme-assisted circularisation of RNA was harnessed by Yi et al. (2022) and Katrekar et al. (2022) with the hypothesis that increasing the half-life of the gRNA would lead to both an increased target efficiency as well as an increase in the editing duration.

**Figure 3:**
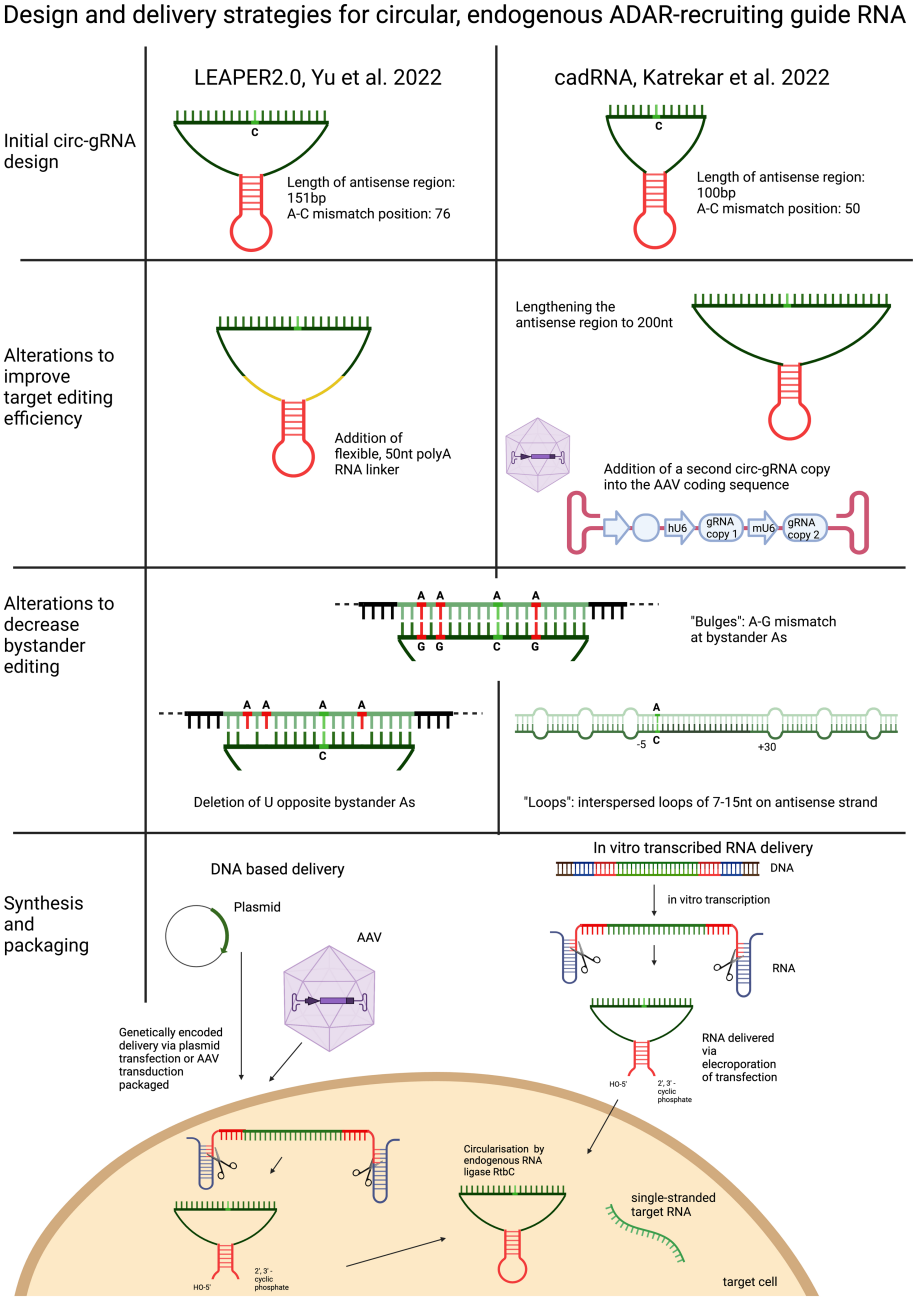
Overview and comparison of the utilisation of circularised guide RNAs. Both circular gRNA are generated with the help of autocatalytically active twister ribozymes and vary in the length of their antisense domain. To improve target efficiency, the antisense region can be lengthened, or the “dose” of the gRNA increased, or the structure of the gRNA made more flexible by including a linker sequence. To decrease bystander editing, deletions, loops and A-G mismatches are used to disrupt perfectly paired gRNA. Circular gRNA can be delivered as DNA or RNA.

Yi et al. based their circularised ADAR-recruiting gRNA (circ-arRNA) on their previously described arRNA, LEAPER. Like in the original design, the target specific antisense region of LEPAER2.0 was 151 nt long, with the A-C mismatch placed at the centre of the sequence and no chemical modifications (Figure 3). To increase editing efficiencies for target sequences that showed no or even reduced benefit from the circ-arRNA (as was the case for *MALAT1* and *KRAS*), a 50 nt, flexible polyAC linker (AC50) was included to increase the structural flexibility of the circ-arRNA. This circ-arRNA_AC50 showed increased editing efficiencies across all target sites. Editing efficiency was tested for 20 sites across 9 endogenous target genes in HEK293T cells. On average, editing efficiency increased by 2.3 for circ-arRNA and by 3.1 for cir-arRNA_AC50 across all target sites when compared to the linear arRNA LEAPER design. Sustained editing >13 days was shown when targeting the *PPIA* transcript in HEK293T cells via transfection. When transduced with AAV containing cir-arRNA, HEK293T, primary human hepatocyte cell line and cerebral organoids showed long term, sustained editing over 9 days, whereas the linear counterparts did not elicit significant editing. *KRAS* was one of two sites that exhibited initial editing efficiencies below that of linear arRNA. Yet over 7 days, editing rates with linear arRNA fell precipitously, while circ-arRNA exhibited continuous, stable editing, resulting in more efficient cumulative editing by circ-arRNA. Similarly, when targeting a reporter plasmid, the expression of arRNA was still detectable at high levels on day 21, whereas the linear arRNA counterpart was not detectable at day 21. While circ-arRNAs produced sustained, high editing efficiencies, they also exhibited pronounced bystander editing along the length of their antisense region. To reduce these bystander edits, the base flipping mechanism required to access ADARs catalytic centre was found to be hindered by deleting the Us opposite the adenosines prone to bystander editing. Different numbers of base deletions were strategically placed across the antisense regions. The cir-arRNA with 14 deletions in the antisense region as well as a flexible AC50 linker was shown to eliminate all bystander edits while retaining a 60% on-target editing efficiency across 8 target sites of the endogenous *PPIA* transcript. A cir-arRNA with a AC50 linker and 4 deletions opposite unwanted adenosines showed a 70% editing rate in a clinically relevant p53 nonsense mutation as well a restoration of full-length protein expression in p53−/− HEK293T cells. Of note, even though the editing rate of cir-arRNA_AC50 (not carrying deletions) was much higher than that of the construct with the deletions, the functional recovery was higher despite the lower editing rate, likely due to the low rate of bystander editing.

To optimise their previously described adRNA, Katrekar et al. undertook a screening of linear adRNA of 100 and 200 nt with and without different ADAR-recruiting domains (GluR2 and Alu recruiting domains as well as the stabilising U6 + 27 cassette) as well as circular adRNA (cadRNA) in HEK293T cells using the 3’ UTR of the *RABA7* transcript as a target. Once again, a positive correlation was seen between gRNA length and on-target editing efficiency, with a linear arRNA of 200 bp resulting in a 1.6-fold increase in on-target editing efficiency compared to the linear 100 bp adRNA counterpart. While addition of ADAR recruitment domains resulted in an only marginal increase in editing efficiency compared to the simple 100 bp linear guide RNA, addition of the stabilising U6 + 27 cassette resulted in a 2-fold increase in target editing and circularisation of the adRNA resulted in a 3.5-fold on-target editing improvement over the linear adRNA. ADAR1 recruitment was confirmed by knockdown of ADAR1 and concomitant abrogation of editing efficiency. To decrease bystander editing, Katrekar et al. employed two strategies (Figure 3): (1) A-G mismatches were introduced opposite all non-target adenosines (cadRNA.bulges) and (2) Introduction of 8 bp loops. While the cadRNA bulges eliminated bystander editing, they also led to a 50% drop in the on-target editing efficiency. By contrast, inclusion of 8 bp loops positioned 5 bp upstream and 30 bp downstream and every 15 bp after that along the antisense domain substantially reduced bystander editing while retaining on target efficiency. CadRNA was tested *in vitro* for its ability to target the coding sequence and 3’ UTR sequences of eight transcripts in HEK293T cells. Twenty-four hours after transfection, robust editing up to 40% was shown in almost all target transcripts with the exception of *TARBDP*, which showed very little editing. The highest editing (90%) was shown when K562 cells were electroporated with genetically encoded cadRNA targeting the 3’UTR of the *RABA7* transcript. These editing rates were similarly high (70% editing) when electroporating the cell line with *in vitro* transcribed RNA (IVT RNA).

Both Yi et al. and Katrekar et al. investigated transcriptome-wide off-target editing of cadRNA/circ-arRNA and compared these with transcriptome- wide off-target editing of exogenously delivered ADARs. In Yi et al. 17 off-target transcriptome wide edits were seen using circ-arRNA, whereas overexpression of exogenous ADAR led to >16,000 transcriptome-wide off-target effects. Katrekar et al. found similar results: With enzyme overexpression 10^3–10^4 off targets edits were routinely observed, this decreased by 2–3-fold when using cadRNAs. Both groups noted cadRNA and circ-arRNA did not lead to a change in mRNA expression levels, indicating the there was no RNA interference mechanism at play.

**Figure 4:**
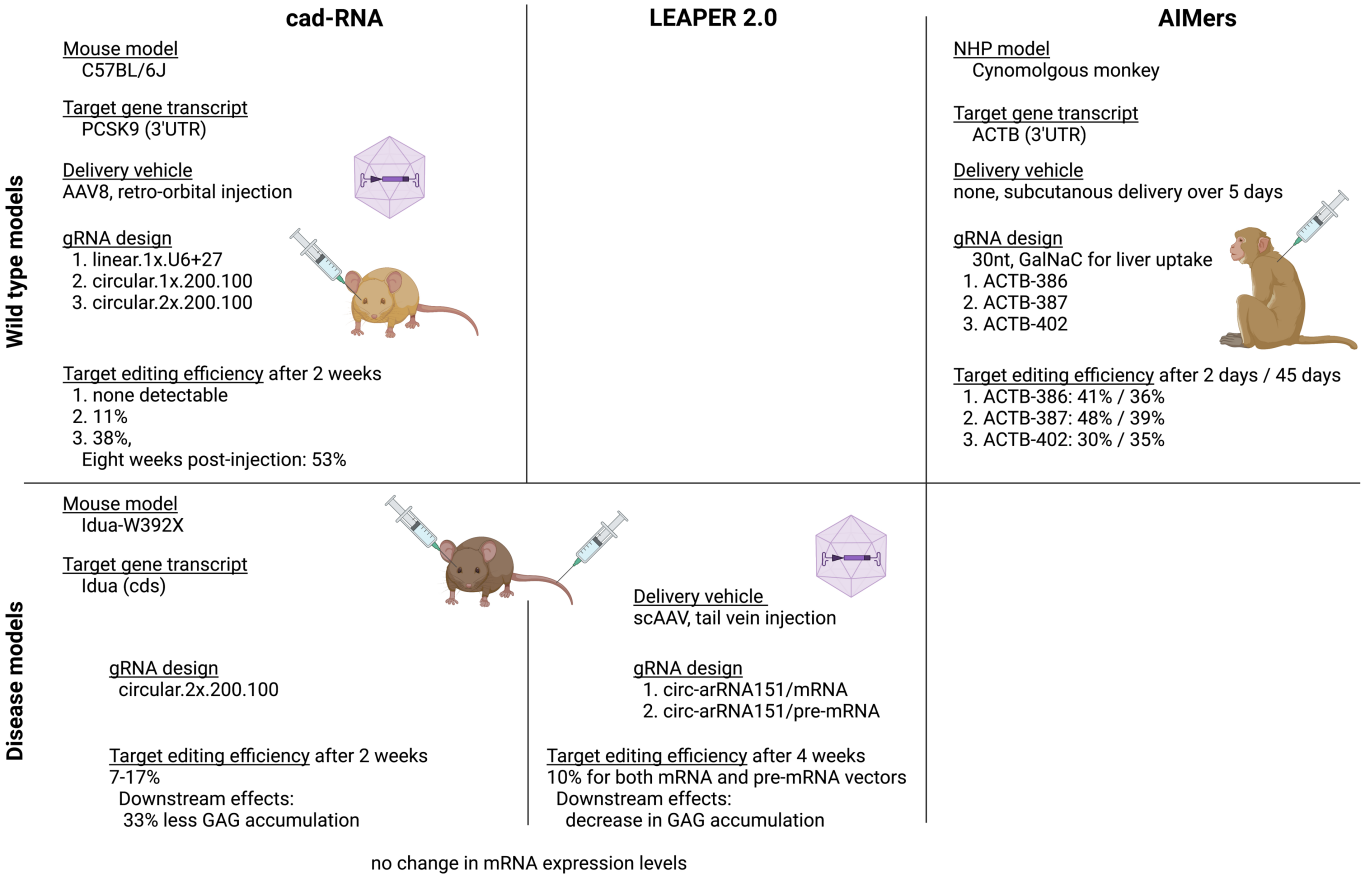
Summary of in-vivo results achieved with circularised and chemically stabilised gRNA. Two wild-type and one disease model were used to evaluate the in-vivo efficacy of endogenous ADAR recruitment. All studies showed sustained on-target editing over a minimum of 2 and a maximum of 8 weeks.

The *in vivo* results generated in both these studies were the first to show sustained editing of a disease-causing mutation using endogenous ADAR (Figure 4). Katrekar et al. first targeted the 3’UTR of the endogenous *PCSK9* gene transcript in the liver of a wild-type C57BL6J mouse model. The best performing linear gRNA with a U6 + U27 cassette was compared with two different cadRNA, one encoding a single copy of the gRNA, while the other encoded two copies of the gRNA. After 2 weeks, the linear gRNA produced no editing, while the two cadRNA exhibited 11 and 38% editing, respectively. Eight weeks post-injection, the editing rates for the cadRNA containing two copies was even higher at 53%. Both Yi et al. and Katrekar et al. used the same mouse model of Mucopolysaccharidosis (MPS) Type I (Hurler syndrome) with the pathogenic nonsense variant W392X in the *Idua* gene to show transcript editing efficiency of cadRNA/circ-arRNA. Both studies used a genetically encoded cadRNA/circ-gRNA packaged in scAAV or AAV8, respectively, to target the liver. Yi et al. showed a 10% targeted editing rate at 4 weeks post injection while Katrekar et al. showed a 7–17% correction of the premature stop codon 2 weeks post-injection. On a protein level endpoint both studies detected a decrease in GAG accumulation (Katrekar et al. measured a 33% decrease) as an indirect marker for partial restoration of alpha-L-iduronidase activity (Figure 3).

#### Proof of principle study using for short ASO to recruit endogenous ADAR in a non-human primate model

**Figure 5:**
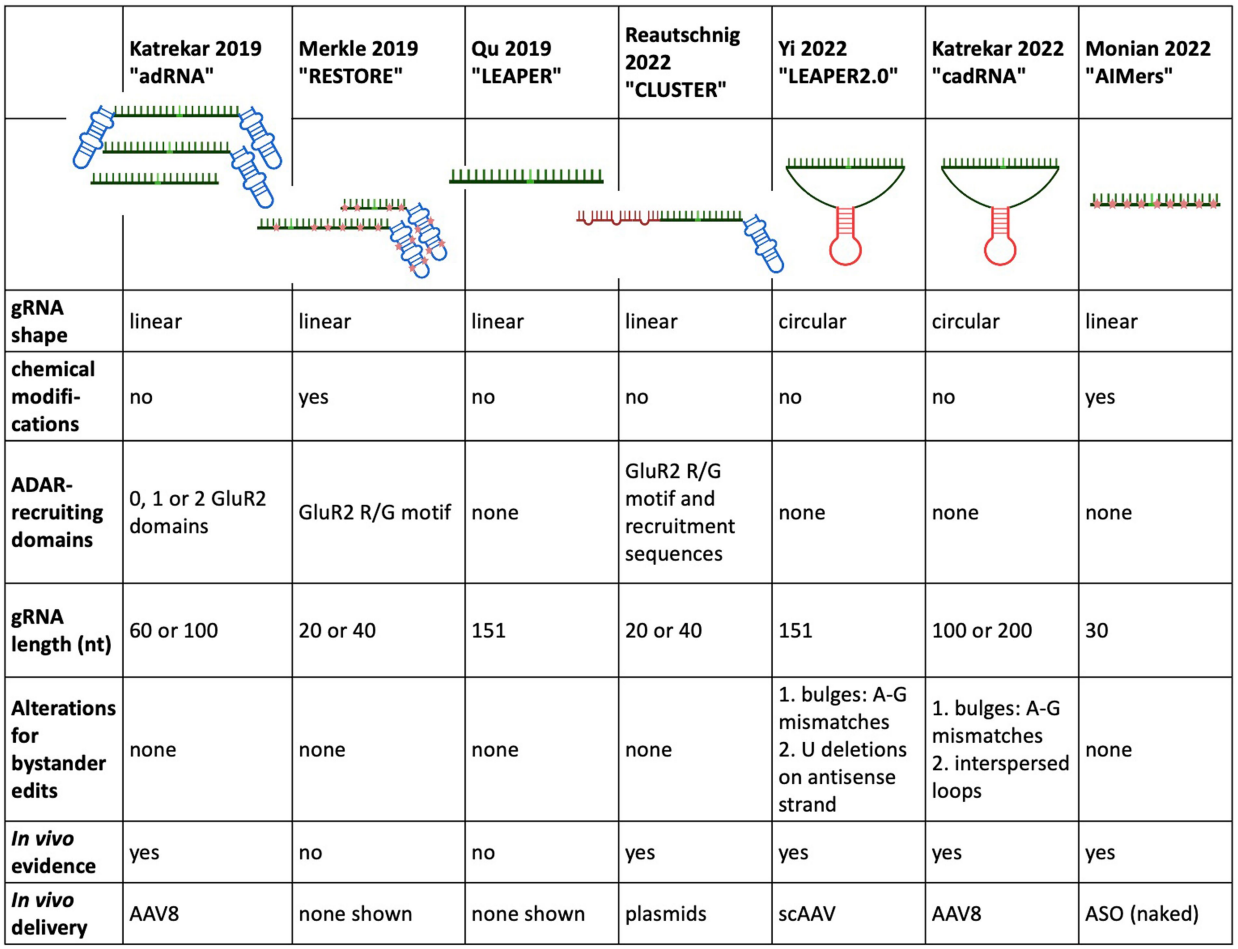
Overview of the design and development of ADAR recruiting guide RNAs.

In the first study demonstrating endogenous ADAR recruitment in a non-human primate (NHP) animal model, Monian et al. used fully chemically modified, short, 30 nt antisense oligonucleotides (AIMers) for endogenous ADAR recruitment. Optimisation of the chemical modifications of the AIMers showed that in addition to the selectively placed 2′ ribose modifications 2’deoxy-, 2’fluoro and 2’-O-methyl, a phosphorothioate (PS) backbone increased target editing activity. PS backbone chirality also influenced editing activity with left-handed (Sp) PS modifications increasing editing efficiency, while right-handed (Rp) PS chirality decreased editing efficiency. Modifications that preferentially enhanced ADAR1 rather than ADAR2 were selected since ADAR1 is expressed ubiquitously at higher levels than ADAR2 and thus may be considered a more therapeutically relevant recruitment target than ADAR2. In addition to optimising chemical modifications for editing activity, an N-acetylgalactosamine (GalNAc) modification was included to improve AIMer delivery to hepatocytes via asialoglycoproteins receptor (Nair et al., 2017). The use of these short and chemically modified AIMers circumvented the need for packaging the gRNA into an ancillary delivery vehicle such as a lipid nanoparticle or AAV. For testing the optimised AIMers *in vivo*, three different, 30 nt GalNAc AIMers were used to target a UAG sequence in the 3’UTR of the *ACTB* transcripts in the liver of cynomolgus monkeys. Six animals were dosed with subcutaneous injections once a day for 5 days. Two days post final injection, all AIMers showed substantial editing ranging from 30 to 50%. At day 45 post injection, the editing persisted and ranged from 35 to 39% (Figure 4). While the editing rates of the initially best performing ADARs decreased over time, the editing rate of the AIMer with the lowest initial editing efficiency of 30% showed a small increase in editing efficiency to 34% post injection, highlighting the importance of gathering longitudinal editing data. No bystander editing or hepatotoxicity was observed and off-target editing at predicted sites was <5%. An overview of the different gRNA designs used to recruit native ADAR is given in Figure 5.

#### Investigating the feasibility of targeting the pre-mRNA with RNA editing

The feasibility of targeting pre-mRNA with RNA editing has been investigated in several studies. Apart from determining which guide RNA is most effective, the ability to target the pre-mRNA would broaden the RNA editing scope to intronic and splice site mutations, which currently present important therapeutic targets in DNA editing and ASO therapy (Maeder et al., 2019; Cideciyan et al., 2021; Musunuru et al., 2021). Furthermore, it would expand RNA editing to target exonic mutations adjacent to splice donor and splice acceptor site, which have been shown to cause both missense and splicing defects (Hodges and Rosenberg, 1989).

Localisation of the components required for editing (endogenous ADAR and gRNA) is an important indication of the feasibility of pre-mRNA targeting. Both Yi et al. and Reautschnig et al. demonstrated gRNA localisation to both the cytoplasm and the nucleus, albeit in different proportions. While Yi et al. found the circ-arRNA to localise to both cellular compartments in equal proportions, Reautschnig et al. found the CLUSTER 16p8 gRNA predominantly localised to the nucleus when transfected. These findings suggest that RNA editing takes place at least partially in the nucleus. The nuclear localisation of ADAR1p110 (and ADAR2) underscores the probability of this assumption. Reautschnig et al. compared the editing yields of a pre-mRNA gRNA to those of a gRNA targeting the mRNA in the coding sequence close to an exon/intron border of three endogenously expressed genes in HEK293FT cell lines. For the *GUSB* target transcript, there was no difference in editing rates between the two gRNAs. For the other two targets, *GPI* and *NUP43*, the pre-mRNA gRNA was found to be one-third and one half as effective as a gRNA targeting mature mRNA. The Wei group investigated pre-mRNA and mRNA gRNA targeting the coding sequence of in the *IDUA* transcript both *in vitro* and *in vivo* (Qu et al., 2019). In a primary fibroblast line of a patient with Hurler syndrome patient, the gRNA targeting the pre-mRNA outperformed the gRNA targeting the mature mRNA. The same held true when the two arRNA were electroporated into another primary fibroblast cell line of a patient with an *IDUA* mutation, where the arRNA targeting the pre-mRNA showed an editing rate of nearly 30%. When tested *in vivo* on the *Idua* W392X mouse model the circ-arRNA/pre-mRNA showed the same target efficiency of 10% as achieved with the circ-arRNA/mRNA. These results were confirmed when looking at IDUA enzyme activity and decrease in GAG accumulation, which both gRNA exhibited in equal measure. In a particularly interesting study, Katrekar et al. used a pre-mRNA targeting gRNA to correct a G > A point mutation located in the last nucleotide of exon 4 of the *OTC* gene transcript of the *spf^ash^* mouse. Due to its location, the G > A mutation was shown to lead to the production of a mutant protein as well as missplicing by read-through of the splice donor site (Hodges and Rosenberg, 1989). A 4.6–8.2% correction in the pre-mRNA transcripts as well as a reduction in the incorrectly spliced product was observed. In the correctly spliced *OTC* mRNA, a high edited fraction (4.6–33.8%) was seen.

### Delivery of RNA structures to photoreceptor cells

For RNA therapeutics to be effective, achieving delivery to the target cell type is a critical step. Whereas RPE cells lend themselves to oligonucleotide uptake by having phagocytic and endocytic capabilities (Storm et al., 2020), entry into photoreceptor cells is more challenging. As RNA structures are functional in the cytoplasm, direct delivery of RNA may be desirable as it avoids the need to overcome the nuclear membrane barrier. Such delivery of small RNA structures appears to be highly viable in the retina with transfer of antisense oligonucleotides (AON) proving very effective. Intravitreal delivery of an AON targeting the mouse *Ush2a* gene was detected in photoreceptor cells up to 259 days post-injection, which was associated with desired exon 12 skipping (Dulla et al., 2021). Developed by ProQR, clinical trials are now ongoing to deliver an AON for skipping the human equivalent region, exon 13 (NCT05085964, NCT05176717, NCT05158296) following encouraging results from a similar AON therapeutic targeting the *CEP290* gene (Russell et al., 2022). These data indicate that small RNA structures can be delivered safely to the vitreous and make their way to the photoreceptor cells where they appear to survive and maintain activity for many weeks. Other chemically modified small RNAs have also been developed and delivered *in vivo* by intravitreal injection with promising pan-retinal survival up to 9 days post-injection (Taniguchi et al., 2020). ADAR-mediated RNA editing has been achieved with chemically modified short RNAs (Merkle et al., 2019; Monian et al., 2022) and though yet to be tested in the retina, the ProQR AON studies offer encouraging signs that translation to the eye may be viable.

However, longer RNA structures may be necessary depending on the treatment strategy, for example, the LEAPER strategy (Qu et al., 2019) uses RNA of 71–91 nucleotides to encourage ADAR recruitment. Delivery of longer RNA structures to the retina has been attempted in different ways. Chemically modified Cy5-labelled mRNA (for translation of GFP) was provided by subretinal or intravitreal injection into 6–7-week-old wild-type mice, either as naked RNA or with a lipofectamine-based transfection reagent (Devoldere et al., 2019). At 24 h post-injection, the mRNA delivered with the transfection reagent by subretinal injection was evident around the injection site in the RPE and photoreceptor cells. By 7 days, the Cy5-labelled mRNA was barely detectable, but GFP expression was apparent in the photoreceptor cells and RPE. No mRNA or GFP expression was evident in eyes that received the naked mRNA. The inclusion of the transfection reagent was also important for mRNA survival following intravitreal injection. At 7 days post-injection, the mRNA was predominantly evident in the ganglion cell layer with some detection in the inner nuclear layer. Sporadic signs of GFP expression were only apparent in the inner nuclear layer. Achieving any delivery of mRNA to the retina and in particular the photoreceptor cells is highly encouraging, but the timeframe of survival and subsequent expression levels are of some concern. RNA editing strategies will likely require sustained presence of the RNA therapeutic and whilst regular repeat treatments of the less invasive intravitreal injection may be viable, it would be preferable to achieve long-term efficacy from a single treatment.

Lipid nanoparticles have been used to carry mRNA encoding reporter proteins (eGFP or mCherry) to the retina (Patel et al., 2019). Initial data indicated subretinal injection of these vectors enabled reporter expression in Müller glia and RPE cells at 24- and 72-h post-injection, which was much reduced by 120 h. Nanoparticles may therefore be an option for treatments targeting the RPE, but the timeframe of the expression profile is again of some concern and delivery of RNA using these carriers has yet to show efficacy in other cell types (Ryals et al., 2020).

An alternative strategy may be to use nanoparticles to carry DNA that enables transcription of the required RNA structures. Whilst various nanoparticle forms provide efficient delivery to the RPE, the polyethylene glycol (PEG)-substituted polylysine (CK30PEG) nanoparticles appear to be a promising form for delivery of therapeutic DNA to photoreceptor cells (Cai et al., 2010; Han et al., 2012, 2015). However, if a DNA element for long-term expression of the RNA structure is required then adeno-associated virus (AAV) is likely to be the vector of choice. Subretinal injection would provide targeted delivery and sustained transgene expression in the photoreceptor cells. Compared to other therapeutic transgenes, RNA structures for enabling endogenous ADAR-mediated editing are relatively short and therefore easy to package. A U6-expression cassette has been used to deliver ADAR-recruiting RNA for editing in mouse muscle (Katrekar et al., 2019) and more recently, the circular ADAR-recruiting guide RNAs discussed previously in this review were packaged in AAV and delivered for editing in the liver (Katrekar et al., 2022). If native ADARs prove to be active in the photoreceptor cells, then delivery of ADAR-recruiting RNA structures may be most effectively provided by AAV.

## Discussion

RNA editing by recruitment of endogenous ADAR is a promising method of harnessing the endogenous posttranscriptional A-to-I editing ADAR enzyme to correct pathogenic SNVs on a transcriptional level without the safety risks of inducing permanent genomic off-target effects. When compared to RNA editing with exogenous ADAR delivery, RNA editing with native ADAR shows three main advantages: (1) Since it requires only the delivery of a nucleic acid sequence and does not require co-delivery of a protein, it harbours potential to decrease immune response to foreign proteins of bacterial origin. (2) It frees up packaging capacity in delivery vehicles such as an AAV, which is limited to 4.7 kB. (3) Lastly, endogenous ADARs achieves target editing with significantly less transcriptome-wide off-target editing compared with strategies relying on ADAR overexpression. Generally, there is a trade-off between achieving high on-target editing with ADAR overexpression and transcriptome wide off-target effects, even when a system is used that only overexpresses the deaminase domain of ADAR (and does not include the dsRBDs). Creating a gRNA that can recruit endogenous ADAR to engage in site-selective RNA editing at high enough levels to show a response at a functional protein level is the goal of gRNA optimisation. Three main caveats should be considered before applying this strategy to a tissue such as the retina. (1) Target tissues and cell types within those target tissues must express ADAR enzymes for editing to occur. Since many of the more recently described ADARs have been optimised for recruitment of the ubiquitously expressed ADAR1p110, the inquiry into ADAR expression needs to establish not only if ADAR expression is present but if it is present at sufficient levels for editing to occur since the gRNA:targetRNA duplex is in competition with natural substrates of endogenous ADARs. (2) Apart from the self-evident sequence limitation that RNA editing with endogenous ADAR can only target G > A SNVs, the target is also limited by the sequence preference of native ADAR, which makes editing of 5’-GAN-3′ motifs particularly inefficient. (3) Thirdly, a feasible delivery to target cells must be considered. Chemically modified gRNA cannot be genetically encoded and packaged into a viral vector and delivery of a naked chemically modified gRNA is only feasible if the gRNA is short. If ADAR expression is low in the target tissue, if the target sequence has a 5′-G and if the target base requires cytidine rather than adenine deamination, RNA editing with exogenous ADAR should be considered the more viable option.

In 2017, two groups published gRNA designs containing an invariable R/G GluR2 recruitment domain and a programmable, short, approximately 20 bp antisense domain for the recruitment of full-length ADAR1 and ADAR2. While designed for recruitment of endogenous ADAR, these gRNAs were not yet capable of recruitment without overexpression of ADAR from a plasmid or a cell line under a strong CMV promotor. Several years later, Katrekar et al. used this basic design and varied the number of R/G GluR2 recruitment domains, length of antisense region and A-C mismatch position. It was shown that with an extension of the antisense domain to at least 60 bp, a recruitment of endogenous ADAR was possible, with or even without the addition of recruitment domains. This was the first indication of a positive correlation between length of the antisense region and target efficiency. These results were replicated *in vivo*, where an antisense region was able to elicit significant, but low editing. Overall, this study showed that recruitment of endogenous ADAR was possible with an antisense domain of at least 60 bp, but the approach was limited due to low target efficiency and was far outperformed by approaches utilising ADAR overexpression, in particular overexpression of the hyperactive ADAR (E488Q) mutant, with full length ADAR or just the deaminase domain. In the same year, two methods were established that enabled efficient gRNA recruitment *in vitro*, LEAPER and RESTORE. LEAPER was able to show editing rates up to 80% *in vitro* by lengthening the antisense domain to 150 bp and beyond, but was unsurprisingly hampered by significant bystander effects, given ADARs propensity for deamination of perfectly paired dsRNA. RESTORE showed encouraging editing rates *in vitro* using the original GluR2 design not by lengthening the antisense domain, but by fully chemically modifying the invariant and the antisense domain of the gRNA and thus increasing the binding affinity of the gRNA (Bennett et al., 2017). These studies convincingly showed that both lengthening of the antisense region as well as chemical stabilisation of the gRNA can lead to higher on-target efficacy. LEAPER demonstrates the trade-off between high on-target efficacy and bystander edits and highlights the need of modification of a long gRNA design to reduce bystander edits, for example through the induction of A-G mismatches. Variability in editing efficiency across different cell lines was thought to be at least in part attributable to cell-line specific ADAR expression or the expression of any RNA editing inhibitors like ADAR3.

Notably, neither of these studies showed application of their gRNA designs *in vivo*. Several years later, the Stafforst group further developed their RESTORE gRNA design and modified it to increase target efficiency without the need for chemical modification of the gRNA, but by adding a cluster of at least three RS distal to the antisense domain. In contrast to simply extending the antisense binding domain, the RS can be flexibly chosen to exclude bystander-prone adenosines, while still strengthening the binding of the target through increasing the gRNA length. By eliminating the need for chemical modification, Reautschnig et al. ensured that the gRNA can be delivered in a genetically encoded way, which might be particularly relevant for the retina since the efficacy of non-viral delivery vehicles (e.g., lipid nanoparticles) for chemically modified mRNA have not yet been established in this setting. In an exciting new development, circularisation of the LEAPER arRNA mark another increase in on-target editing efficiency for RNA editing with endogenous ADAR. It should be noted that throughout both Yi et al. and Katrekar et al. while by and large circularisation dramatically improved editing efficiencies, the target efficiencies varied greatly between targets and cell lines. Within the same target gene transcript, editing results varied greatly, which perhaps points to the sequence preference and structural selectivity that native ADAR exhibits. However, even with low initial target efficiencies, it could be argued that due to the increased stability and longer half-life, a circularised gRNA could still outperform an equivalent linear version due to sustained editing over time, an effect essential for RNA based therapies. Circularised gRNA also showed robust and sustained editing rates in both wild-type and disease mouse models. From a delivery perspective, both groups used genetically encoded gRNA packaged into an AAV, a highly relevant mode of delivery for IRDs. Future modifications of circular gRNA might include further stabilisation by chemical modification or addition of recruitment segments. A further exciting development was shown in Monian’s et al. proof-of-concept study demonstrated very promising, high target efficiency both 2 days post AIMer delivery (up to 50%) and over 1 month time period (up to 40%) without bystander and with minimal off-target effects. It remains to be seen if these results can be replicated on a protein and phenotype level targeting a coding sequence in a disease-causing mutation. Katrekar et al. experienced a drop in efficacy when moving from a wild type non-coding sequence to disease causing transcript target. For retinal application, these short, 30 nt AIMers are of particular interest since they hold promise of a straightforward intravitreal delivery, whereas the length of previous chemically modified gRNA such as RESTORE would require delivery in a non-viral packaging vehicle such as a lipid nanoparticle. While these are promising, their primary target has to date been the liver, an organ that by its nature is designed for optimal uptake (similarly to the RPE). It should be noted that all reviewed trials looking for therapeutic efficacy of RNA editing with endogenous ADAR *in vivo* have been designed to target the liver, and therapeutic efficiacy in other organs has yet to be established. While there have been reports of using Mini-dCas13X for Duchenne muscular dystrophy (Li et al., 2023), RNA base editors recruiting endogenous ADAR for diseases with target tissues outside the liver have yet to be tested. The retina is an ideal therapeutic target tissue candidate due to its accessibility, the optimised subretinal surgical application from previous gene therapy trials and the need for innovative therapies for monogenic diseases. *In vivo* trials showing proof of principle targeting different therapeutic target transcripts in varied target tissue will be needed to fully evaluate the therapeutic potential of RNA editing with endogenous ADAR.

## Conclusion

RNA editing with endogenous ADARs is a highly specific, reversible method of correcting G > A polymorphisms on a transcript level that has recently undergone several promising steps toward potential clinical application. Despite these benefits, editing via recruitment of endogenous ADAR has been hampered by low on target editing efficiency when compared to overexpression of exogenous ADAR. This could be due both to the availability of native ADAR and the inherent instability of linear gRNA as it is exposed to exonuclease mediated degradation. Methods to tackle low target editing efficiency by lengthening the antisense region have often hampered by high bystander editing whereas chemical modifications of long gRNA present the drawback of not being genetically encodable. Circularisation of the gRNA as well as chemical modification of a short AIMer both stabilise the guide RNA half-life and are the first to present a step toward viable editing with endogenous ADARs *in vivo*. Editing is restricted to adenosines in dsRNA motifs containing preferred neighbouring nucleotides in tissues with sufficient levels of endogenous ADAR activity to support ADAR recruitment by c gRNAs. The maximal development of both the exogenous and endogenous ADAR is needed to broaden the toolset of RNA editing to all for transition mutations including those that have non-preferred neighbouring nucleotides or exhibit non-advantageous secondary structures. Before RNA editing with endogenous ADAR can be employed in the retina, for example in photoreceptors, levels of ADAR1 and ADAR2 must be elucidated in the retinal target cells. Ultimately, feasibility will depend on the expression level of endogenous ADAR within retinal target cells as well as the identification of a suitable delivery strategy that will enable stable gRNA expression as well as photoreceptor uptake. Given the ubiquitous expression profile of ADAR1 and the encouraging developments of ADAR-recruiting RNA elements to date, there is great potential to employ an endogenous RNA editing strategy for correction of G > A mutations in IRDs.

## Author contributions

JSB, MM, and REM contributed to the conception and design of this review. JSB wrote the first draft of the manuscript and undertook data curation and formal analysis. MM wrote one section of the manuscript. REM and MDF acquired funding for this review. All authors contributed to the manuscript revision, read, and approved the submitted version.

## Funding

This research was funded by St Cross Mabel Churn Scholarship (JSB) and the NIHR Oxford Biomedical Research Centre (REM, MDF, MEM). The views expressed are those of the authors and not necessarily those of the NHS, the NIHR or the Department of Health.

## Conflict of interest

The authors declare that the research was conducted in the absence of any commercial or financial relationships that could be construed as a potential conflict of interest.

## Publisher’s note

All claims expressed in this article are solely those of the authors and do not necessarily represent those of their affiliated organizations, or those of the publisher, the editors and the reviewers. Any product that may be evaluated in this article, or claim that may be made by its manufacturer, is not guaranteed or endorsed by the publisher.

## Footnotes

^1^ https://www.proteinatlas.org
